# Transposable Elements and DNA Methylation Create in Embryonic Stem Cells Human-Specific Regulatory Sequences Associated with Distal Enhancers and Noncoding RNAs

**DOI:** 10.1093/gbe/evv081

**Published:** 2015-05-07

**Authors:** Gennadi V. Glinsky

**Affiliations:** ^1^Institute of Engineering in Medicine, University of California, San Diego; ^2^The Stanford University School of Medicine, Department of Surgery, Stanford, California

**Keywords:** retrotransposons, repetitive elements, primate evolution, human ESC, pluripotent state regulators, NANOG, POU5F1 (OCT4), CTCF, methyl-cytosine deamination, LTR7 RNAs, L1 retrotransposition, LINE, LTR, LTR7/HERVH, evolution of Modern Humans

## Abstract

Despite significant progress in the structural and functional characterization of the human genome, understanding of the mechanisms underlying the genetic basis of human phenotypic uniqueness remains limited. Here, I report that transposable element-derived sequences, most notably LTR7/HERV-H, LTR5_Hs, and L1HS, harbor 99.8% of the candidate human-specific regulatory loci (HSRL) with putative transcription factor-binding sites in the genome of human embryonic stem cells (hESC). A total of 4,094 candidate HSRL display selective and site-specific binding of critical regulators (NANOG [Nanog homeobox], POU5F1 [POU class 5 homeobox 1], CCCTC-binding factor [CTCF], Lamin B1), and are preferentially located within the matrix of transcriptionally active DNA segments that are hypermethylated in hESC. hESC-specific NANOG-binding sites are enriched near the protein-coding genes regulating brain size, pluripotency long noncoding RNAs, hESC enhancers, and 5-hydroxymethylcytosine-harboring regions immediately adjacent to binding sites. Sequences of only 4.3% of hESC-specific NANOG-binding sites are present in Neanderthals’ genome, suggesting that a majority of these regulatory elements emerged in Modern Humans. Comparisons of estimated creation rates of novel TF-binding sites revealed that there was 49.7-fold acceleration of creation rates of NANOG-binding sites in genomes of Chimpanzees compared with the mouse genomes and further 5.7-fold acceleration in genomes of Modern Humans compared with the Chimpanzees genomes. Preliminary estimates suggest that emergence of one novel NANOG-binding site detectable in hESC required 466 years of evolution. Pathway analysis of coding genes that have hESC-specific NANOG-binding sites within gene bodies or near gene boundaries revealed their association with physiological development and functions of nervous and cardiovascular systems, embryonic development, behavior, as well as development of a diverse spectrum of pathological conditions such as cancer, diseases of cardiovascular and reproductive systems, metabolic diseases, multiple neurological and psychological disorders. A proximity placement model is proposed explaining how a 33–47% excess of NANOG, CTCF, and POU5F1 proteins immobilized on a DNA scaffold may play a functional role at distal regulatory elements.

## Introduction

Identification of the genomic regulatory sequences contributing to the development of species-specific phenotypic features during primate evolution remains a significant challenge with important fundamental and translational implications. Determination of the molecular mechanisms underlying the genetic basis of human phenotypic uniqueness represents a particularly challenging task. Despite remarkable progress in understanding the structural–functional diversity, transcriptional and regulatory complexities, and biological significance of nonprotein-coding genomic regions, protein-coding genes remain the main focus of the experimental and theoretical analyses of human-specific genetic traits. Accelerated evolution of a selected set of protein-coding genes involved in various aspects of nervous system development and biology has been demonstrated (Dorus et al. 2004; Zhang et al. 2011). Increased rates of protein evolution are most prominent for genes regulating brain size and behavior within the evolutionary lineage leading from ancestral primates to humans (Dorus et al. 2004). Recent experiments have identified microRNA-associated regulation of brain gene expression as one type of human-specific regulatory mechanism that is undergoing accelerated evolution (Somel et al. 2011). It has been suggested that human-specific histone methylation signatures at defined transcription start sites in prefrontal neurons may play an important biological role in the emergence and functions of human-specific gene expression networks in the brain (Shulha et al. 2012).

The increase in the content and quality of genome and transcriptome sequence databases has facilitated comparative genomics studies focused on identification of primate-specific genes in the human genome. Taken together, these reports have identified a few hundred candidate primate-specific genes for follow-up functional studies, demonstrated insertions of interspersed repeats in exons of 92% of the identified primate-specific genes, documented the placement of sequences derived from transposable elements (TEs) in 53% of primate orphan genes, and highlighted the involvement of nonduplicated pericentromeric and subtelomeric regions of the human genome in the genesis of primate-specific genetic elements (Tay et al. 2009; Toll-Riera et al. 2012). Early pioneering work has demonstrated that TEs have contributed to the origin of a substantial fraction of human regulatory sequences (Jordan et al. 2003). Analysis of DNase I hypersensitivity sites in more than 40 distinct human cell types demonstrated that 63% of primate-specific open chromatin regions were in TEs, indicating that TEs have contributed hundreds of thousands of novel regulatory elements to the primate lineage (Jacques et al. 2013). However, systematic efforts aiming at genome-wide identification and characterization of human-specific regulatory sequences such as transcription factor (TF)-binding sites are lacking.

Repetitive and repeat-derived DNA sequences, including TEs, may account for up to two-thirds of the human genome (De Koning et al. 2011). TEs contribute to a multitude of genomic regulatory functions and have been identified as a rich source for the creation of new species-specific TF-binding sites in mammals (Wang et al. 2007; Bourque et al. 2008; Kunarso et al. 2010). Most recently, TEs have been implicated in the origin, regulation, and specification of long noncoding (lnc) RNAs (Kelley and Rinn 2012; Kapusta et al. 2013). It has been documented that TEs are embedded within 83% of human lncRNAs and comprise 42% of lncRNA sequences, which is in sharp contrast to the small TE content within protein-coding genes in the human genome (Kelley and Rinn 2012).

In human cells, retrotransposon activity is believed to be suppressed to restrict the potentially harmful effects of mutations on functional genome integrity and maintenance of genomic stability. Human embryonic stem cells (hESCs) seem markedly different in this regard. Several studies demonstrated that hESCs express mRNAs from both human-specific (L1HS) and older L1 subfamilies of retrotransposons (Garcia-Perez et al. 2007; Macia et al. 2011), and activity of L1 retrotransposition is markedly elevated in hESCs (Wissing et al. 2012). Human endogenous retrovirus subfamily H (HERV-H) RNA expression is markedly increased in hESCs, and an enhanced rate of insertion of HERV-H sequences appears to be associated with binding sites for pluripotency TFs and lncRNAs (Kelley and Rinn 2012; Santoni et al. 2012; Xie et al. 2013). These data suggest that TEs may contribute to the creation of human-specific regulatory sequences associated with a pluripotent stem cell phenotype. Activation of retrotransposons and lineage-specific repeat-driven dispersion of CCCTC-binding factor (CTCF)-binding sites has produced species-specific expansions of CTCF binding in the genomes of rodents, dogs, and opossums (Schmidt et al. 2012). Importantly, this evolutionary engineering of new CTCF-binding events appears to create binding sites that function as chromatin domain insulators and transcriptional regulators (Schmidt et al. 2012). Surprisingly, there was no evidence found indicating the enrichment of CTCF-binding events within species-specific repeats in the human or macaque genomes (Schmidt et al. 2012).

Here, I report the identification and initial characterization of 4,094 candidate human-specific regulatory loci, 3,803 of which harbor putative TF-binding sites in hESC. Candidate human-specific regulatory elements are preferentially located in hESC within the matrix of hypermethylated DNA segments and appear engaged in selective and site-specific binding of the critical regulatory proteins NANOG (Nanog homeobox), POU class 5 homeobox 1 (POU5F1) (OCT4), CTCF, and Lamin B1. Sequences of hESC-specific TF-binding sites do not intersect with any chains in the mouse, rat, chimp, gorilla, orangutan, gibbon, rhesus, and marmoset genomes. This analysis reveals exclusive-to-primates DNA editing mechanisms involving DNA methylation at selected cytosine residues within LTR7 sequences coupled with methyl-cytosine deamination events that are overrepresented in the human genome and appear involved in the creation of regulatory sites with novel protein-binding specificities.

## Materials and Methods

### Data Sources

Solely publicly available data sets and resources were used for this analysis as well as methodological approaches and a computational pipeline validated for primate-specific gene discovery (Kent 2002; Schwartz et al. 2003; Tay et al. 2009). The analysis is based on the UCSC LiftOver conversion of the coordinates of human blocks to nonhuman genomes using chain files of precomputed whole-genome BLASTZ alignments with a minMatch of 0.95 and other search parameters in default setting (http://genome.ucsc.edu/cgi-bin/hgLiftOver, last accessed May 20, 2015). Extraction of BLASTZ alignments by the LiftOver algorithm for a human query generates a LiftOver output “Deleted in new,” which indicates that a human sequence does not intersect with any chains in a given nonhuman genome. This indicates the absence of the query sequence in the subject genome and was used to infer the presence or absence of the human sequence in the nonhuman reference genome. Human-specific regulatory sequences were manually curated to validate their identities and genomic features using a BLAST (Basic Local Alignment Search Tool) algorithm and the latest releases of the corresponding reference genome databases as of April/May 2013.

Data sets of NANOG-, POU5F1-, and CTCF-binding sites in hESCs were reported previously (Kunarso et al. 2010) and are publicly available. RNA-Seq data sets were retrieved from the UCSC data repository site (http://genome.ucsc.edu/ [last accessed May 20, 2015]; Meyer et al. 2013) for visualization and analysis of cell type-specific transcriptional activity of defined genomic regions. A genome-wide map of the human methylome at single-base resolution was reported previously (Lister et al. 2013) and is publicly available (http://neomorph.salk.edu/human_methylome, last accessed May 20, 2015). The histone modification and TF chromatin immunoprecipitation sequence (ChIP-Seq) data sets for visualization and analysis were obtained from the UCSC data repository site (http://genome.ucsc.edu/, last accessed May 20, 2015; Rosenbloom et al. 2013). Genomic coordinates of the RNAPII-binding sites, determined by the chromatin integration analysis with paired end-tag sequencing (ChIA-PET) method, were obtained from the saturated libraries constructed for the MCF7 and K562 human cell lines (Li et al. 2012). Genome-wide maps of interactions with nuclear lamina, defining genomic coordinates of human and mouse LADs, were obtained from previously published and publicly available sources (Guelen et al. 2008; Peric-Hupkes et al. 2010). The density of TF-binding to a given segment of chromosomes was estimated by quantifying the number of protein-specific binding events per 1-Mb and 1-kb consecutive segments of selected human chromosomes and plotting the resulting binding site density distributions for visualization. Visualization of multiple sequence alignments was performed using the WebLogo algorithm (http://www.weblogo.berkeley.edu/logo.cgi, last accessed May 20, 2015). Consensus TF-binding site motif logos were previously reported (Kunarso et al. 2010; Wang et al. 2012; Ernst and Kellis 2013).

The quantitative limits of proximity during the proximity placement analyses were defined based on the metrics placing human-specific TF-binding sites closer to putative target protein-coding or lncRNA genes than experimentally defined distances to the nearest targets of 50% of the regulatory proteins analyzed in hESCs (Guttman et al. 2011). For each gene of interest, all human-specific NANOG- and CTCF-binding sites were identified and tabulated with a genomic distance between TF-binding sites and a putative target gene that is smaller than the mean value of distances to the nearest target genes regulated by the protein-coding TFs in hESCs. The corresponding mean values for protein-coding and lncRNA target genes were calculated based on distances to the nearest target genes for TFs in hESC experimentally determined by Guttman et al. (2011).

The assessment of conservation of 826 human-specific NANOG-binding sites in individual genomes of three Neanderthals and five Modern Humans was carried out by direct comparisons of corresponding sequences retrieved from individual genomes and the human genome reference database (http://genome.ucsc.edu/Neandertal/, last accessed May 20, 2015). Direct access to the specific Genome Browser tracks utilized for analyses and visualization: http://genome.ucsc.edu/cgi-bin/hgTracks? db=hg18&position=chr10%3A69713986-69714099&hgsid= 393865029_yg7UixUE4a4awjjTahns4KTPkIl1 (last accessed May 20, 2015).

### Statistical Analyses of the Publicly Available Data Sets

All statistical analyses of the publicly available genomic data sets, including error rate estimates, background and technical noise measurements and filtering, feature peak calling, feature selection, assignments of genomic coordinates to the corresponding builds of the reference human genome, and data visualization, were performed exactly as reported in the original publications and associated references linked to the corresponding data visualization tracks (http://genome.ucsc.edu/, last accessed May 20, 2015). Any modifications or new elements of statistical analyses are described in the corresponding sections of the Results. Statistical significance of the Pearson correlation coefficients was determined using GraphPad Prism version 6.00 software. The significance of the differences in the numbers of events between the groups was calculated using two-sided Fisher’s exact and Chi-square test, and the significance of the overlap between the events was determined using the hypergeometric distribution test (Tavazoie et al. 1999).

## Results

### Repetitive Elements are Associated with More than 99% of the Candidate Human-Specific TF-Binding Sites in hESCs

To identify candidate human-specific regulatory sequences, I carried out sequence homology profiling of 205,974 binding sites for NANOG, OCT4, and CTCF proteins detected in hESCs (Kunarso et al. 2010) across human, rodent, and primate reference genome databases. In the hg18 human genome reference database, the LiftOver algorithm (http://genome.ucsc.edu/cgi-bin/hgLiftOver, last accessed May 20, 2015) identified 29,130; 14,003; and 29,018 sequences of 200 bp in length centered at NANOG-, OCT4-, and CTCF-binding sites, respectively, that do not intersect with any chains in the mouse and rat genomes (supplementary data sets S1–S3, Supplementary Material online). Overall, these sequences constitute 33% of all TF-binding events detected in hESCs for NANOG and CTCF and 47% of all OCT4-binding sites (table 1). Similar results were obtained when the analysis was performed employing the hg19 release of the human genome reference database, confirming the consistency of mapping and alignments for these regulatory sequences across databases. These sequences were thus defined as candidate primate-specific TF-binding sites. To determine whether this collection of sequences contains a subset of human-specific regulatory loci, a search for TF-binding sites was conducted that conform to the following criteria: 1) Represent sequences uniquely mapped to a single genomic location in both the hg18 and hg19 releases of the human reference genome database; and 2) do not intersect with any chains in the rodent and primate genomes, including the mouse, rat, chimp, gorilla, orangutan, gibbon, rhesus, and marmoset genomes. This analysis identified 826, 2,386, and 591 human-specific binding events for the NANOG, OCT4, and CTCF TFs, respectively (supplementary data sets S4–S6, Supplementary Material online). Notably, these sets of human-specific regulatory sequences represent 0.9%, 8%, and 0.7% of all binding sites and 3%, 17%, and 2% of primate-specific binding sites for the NANOG, OCT4, and CTCF TFs, respectively (table 1).

**Table 1:** Summary of the Search for Human-Specific TF-Binding Sites

Regulatory Proteins	hESC Genome	Associated with Repeats	Percent within Repeats	Primate-Specific Sites	Human-Specific Sites	Human-Specific Sites within Repeats	Percent within Repeats	LTR/LINE Embedded	Percent within LTR/LINE
NANOG	88,351	13,200	14.9	29,130 (33%)	826 (0.9%)	824	99.8	532	64.4
CTCF	87,883	10,021	11.4	29,018 (33%)	591 (0.7%)	590	99.8	104	17.6
POU5F1	29,740	6,619	22.3	14,003 (47%)	2,386 (8%)	2,383	99.9	116	4.9
RNAPII	30,585	ND	ND	12,012 (39%)	319 (1%)	319	100.0	5	1.6

Note.—NANOG-, CTCF-, and POU5F1-binding sites were determined by the ChIP-Seq method in the H1-hESC line (Kunarso et al. 2010; hg18 database counts); RNAPII-binding sites were determined by the ChIA-PET method in the MCF7 and K562 cell lines (Li et al. 2012; hg19 database counts); ND, not determined.

**Fig. 1.—:**
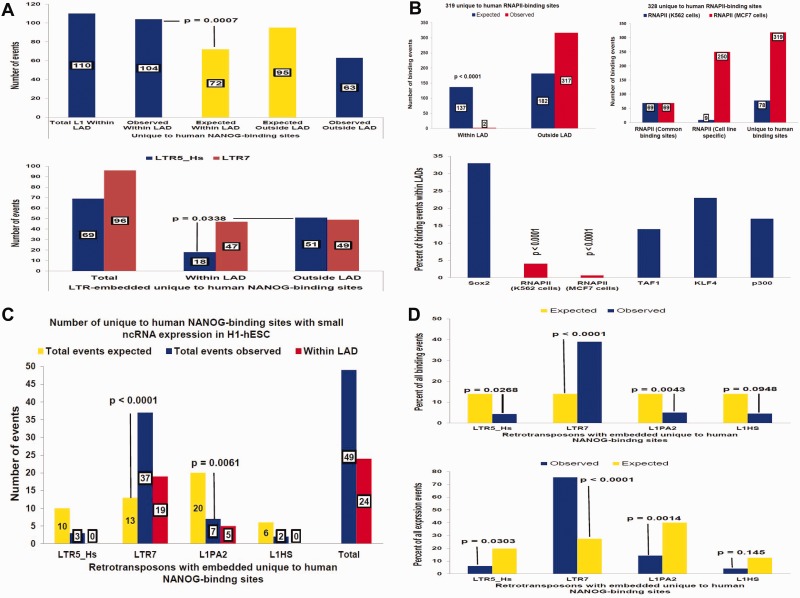
Characterization of genomic features associated with human ESC-specific NANOG- and CTCF-binding sites. Location of full-length L1 TE sequences containing human ESC-specific NANOG-binding sites is enriched within LADs (*A*). In the hESC genome, there are 184 human-specific NANOG-binding sites that are embedded within 167 full-length and 17 truncated L1HS and L1PA2 sequences, 110 of which are located within LADs (*A*, top panel). In total, 104 of 110 (95%) of the L1HS and L1PA2 sequences containing human-specific NANOG-binding sites and located within LADs are preserved as full-length (5,962–6,189 bp) L1 retrotransposons (*A*, top panel), indicating that the conservation of the full-length L1 TE containing human-specific NANOG-binding sites is significantly higher within LADs. In contrast, the majority of LTR5_HS-embedded human-specific NANOG-binding sites is found outside of LADs, whereas LTR7-embedded human-specific NANOG-binding sites are equally distributed within and outside of LADs (*A*, bottom panel). Distinct placement patterns within LADs of 446 human-specific TF-binding sites for five different regulatory proteins (*B*). Note that a significantly higher fraction than expected by chance of RNAPII-binding sites is located outside of LADs (*B*) in contrast to other TF-binding sites. LTR7-derived sncRNAs represent the predominant type of sncRNAs generated by transcriptional activity of the human-specific NANOG-binding sites in hESCs and are transcribed from the loci equally distributed within and outside the LADs (*C*, *D*).

To determine whether repetitive elements contributed to the creation of putative human-specific TF-binding sites, the sequences of 200-bp windows centered at the middle of the TF-binding sites were intersected with the RepeatMasker database track of the University of California Santa Cruz (UCSC) Genome Browser (http://www.repeatmasker.org/, last accessed May 20, 2015). Each overlapping event was tabulated and the numbers of overlaps of each TF’s binding event with specific repetitive elements were calculated. Notably, 99.8% (3,797 of 3,803) of human-specific TF-binding sites were found embedded within repetitive elements, which is significantly higher than the proportion expected by chance (*P* << 0.0001; hypergeometric distribution test). Follow-up analyses indicated that the “99% rule” is not limited to the NANOG-, OCT4-, and CTCF-binding sites and seems to have broad relevance. All human-specific binding events identified for five different regulatory proteins (SOX2, RNAPII [RNA polymerase II], TAF1, KLF4, and p300) mapped within repeat-derived sequences in the reference human genome database (table 1 and fig. 1).

In total, 532, 116, and 104 of the candidate human-specific binding sites for NANOG, OCT4, and CTCF, respectively, were found located within long interspersed nuclear element (LINE) and long terminal repeat (LTR) sequences, suggesting that retrotransposons play a significant role in the creation of human-specific regulatory sequences (supplementary data sets S4–S6, Supplementary Material online). LINE- and LTR-embedded human-specific TF-binding sites constitute 64%, 5%, and 18% of all human-specific binding events for NANOG, OCT4, and CTCF, respectively. LINE and LTR elements appear to show equal contribution to the creation of human-specific TF-binding sites for NANOG, OCT4, and CTCF (supplementary fig. S1, Supplementary Material online). Interestingly, a majority of the LINE- and LTR-embedded human-specific TF-binding events in hESCs are represented by the retrotransposons L1PA2 (196 events), L1HS (64), LTR7 (109), and LTR5_HS (81) that are transcriptionally active in human cells (supplementary fig. S1, Supplementary Material online). Notably, human-specific TF-binding sequences are often embedded within full-length 6-kb L1PA2 and L1HS sequences. For example, 95% of L1PA2-embedded NANOG-binding sites are located within the full-length L1PA2 sequences. Similarly, full-length 6-kb L1HS sequences with less than 1% divergence constitute 77% of all L1HS sequences with human-specific TF-binding sites in hESCs.

Taken together with the recent observations that L1 retrotransposition is elevated in hESCs (Wissing et al. 2012), human embryos (Smith et al. 2014), induced pluripotent stem cells (iPSC) of humans and great apes (Marchetto et al. 2013), and brain tissues from individual humans (Baillie et al. 2011) and earlier reports that mRNAs from both human-specific L1HS and older L1 subfamilies of retrotransposons are expressed in hESCs (Garcia-Perez et al. 2007; Macia et al. 2011), these data support the idea that L1 retrotransposition is continually altering the landscape of human genome, perhaps, contributing to the creation of human-specific regulatory loci. Conclusive experimental evidence of the LTR7/HERVH activation in hESC (Kelley and Rinn 2012; Santoni et al. 2012; Xie et al. 2013; Lu et al. 2014) are consistent with the idea that increased activity of HERVs may contribute to these processes. It will be essential to determine whether the active retrotransposition is continually altering the genome of human germline cells.

### Human-Specific TF-Binding Events Manifest as Distinct Regulatory Patterns in hESCs and Differentiated Cells

One of the most notable features of the candidate human-specific regulatory loci reported in this study is that many of the TF-binding sites active in hESCs are not bound by TFs in differentiated cells. This observation is consistent with the documented essential role of NANOG and OCT4 in the maintenance of the pluripotent state and the restriction of their functions to ESCs. Therefore, a majority of candidate human-specific regulatory sequences identified in this study should be defined as candidate human-specific regulatory loci harboring putative TF-binding sites in hESC. Significantly diminished CTCF-binding in differentiated human cells compared with hESCs (supplementary fig. S1*D*, Supplementary Material online) suggests that the function of a large number of CTCF-binding events is associated with the pluripotent state. Interestingly, loss of CTCF occupancy in differentiated cells appears limited to the human- and primate-specific binding events (supplementary fig. S1*D*, Supplementary Material online), indicating that pluripotency-associated CTCF activity may be mediated by primate-specific binding sites. Recent experimental evidence supports the idea of specialized CTCF-dependent regulatory mechanisms that are critical for the self-renewal and pluripotency properties of hESCs. Balakrishnan et al. (2012) reported on functional analyses of individual CTCF-binding sites in hESCs by demonstrating specialized, site- and target gene-specific CTCF-dependent insulator, enhancer, or repressor activities.

As noted above, NANOG-binding sites in hESCs seem particularly interesting in this context because nearly two-thirds of human-specific NANOG-binding events are embedded within LINE and LTR retrotransposons in contrast to only 5% and 18% binding sites for OCT4 and CTCF, respectively. Consistent with this line of arguments, 34 of the 39 (87%) TF-binding loci embedded within transcriptionally active full-length L1HS retrotransposons (supplementary data set S4, Supplementary Material online) are occupied by NANOG, which is significantly more than expected by chance (*P* = 0.00086). In contrast, only seven full-length L1HS-embedded human-specific TF-binding sites are occupied by OCT4, five of which overlap with NANOG-binding sites. Similarly, only three full-length L1HS-embedded human-specific TF-binding sites are occupied by CTCF, which is marginally significantly less than expected by chance. These conclusions remain statistically valid after accounting for five overlapping sites.

It can thus be concluded that human-specific TF-binding loci appear to operate in hESCs in distinct and exclusive modes compared with differentiated cells by engaging in selective and site-specific binding of the critical regulatory proteins NANOG, OCT4, and CTCF. Given the high diversity of TF-binding sites in mammalian genomes recognizing myriads of DNA elements and the continuing expansion of TEs in the human population, many of these correlations are likely to represent chance events that are unrelated to natural selection. Further functional studies will be required to clarify the biological significance (if any) of the apparent structural differences between hESCs and differentiated cells.

### Candidate Human-Specific TF-Binding Sites Exhibit Distinct Patterns of Transcriptional Activity and Association with Nuclear Lamina

**Fig. 2.—:**
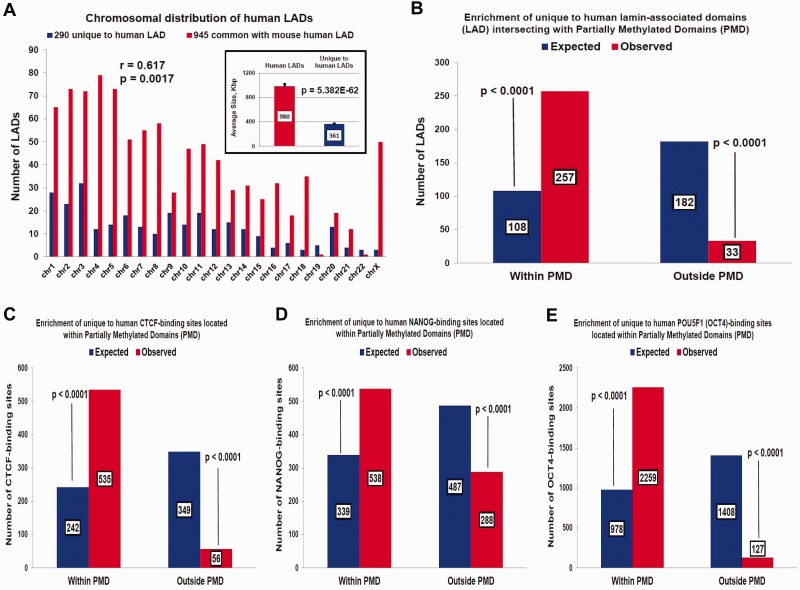
Candidate human-specific regulatory elements are associated with genomic regions that are hypermethylated in hESCs. Human-specific LADs, comprising 21% of all nuclear lamina-associated genomic regions in the human genome, are represented on all human chromosomes, constitute more than 25% of LADs on 50% of human autosomes, are significantly smaller in size compared with all LADs in the human genome (*A*), and manifest highly correlated patterns of chromosomal distributions with 29,018 CTCF-bound and 29,130 NANOG-bound primate-specific TF-binding sites (see also supplementary fig. S3, Supplementary Material online). Genomic coordinates of 4,094 candidate human-specific regulatory loci representing four distinct classes of genomic regulatory elements are enriched within chromosomal regions that are hypermethylated in hESCs compared with differentiated human cells, and are designated as PMDs (*B–E*).

**Fig. 3.—:**
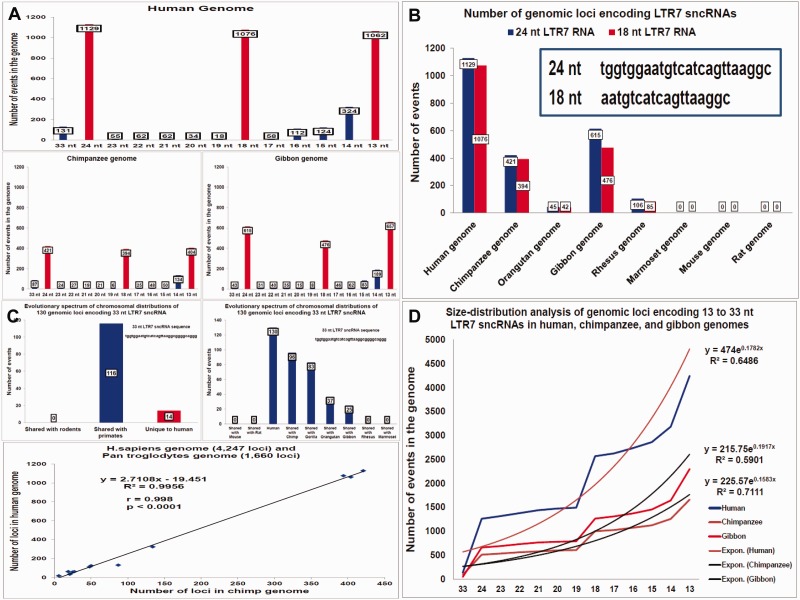
Analysis of LTR7-derived sncRNAs transcribed from human-specific regulatory loci reveals evolutionary conservation of wild-type sequences. Distinct molecular variants of the 33- and 24-nt LTR7 sncRNAs manifest a primate-specific evolutionary spectrum of chromosomal distributions (*A–D*) and highly correlated patterns of chromosomal distributions in the human genome (*r* = 0.865; *P* < 0.0001; supplementary fig. S4, Supplementary Material online). Size distribution analyses in the human and primate genomes of fully conserved LTR7 sncRNA-encoding loci of various lengths revealed essentially identical profiles of genome-wide size distributions of the LTR7 sncRNA-encoding loci in the human, chimpanzee, and gibbon genomes (*A*) with identical 24- and 18-nt sequences representing the predominantly conserved molecular entities (*A*, *B*), which are significantly overrepresented in the human genome compared with the primate and rodent genomes (*A*, *B*, *D*). Panel (*A*) shows results of the size distribution analysis and panel (*D*) shows the linear regression analysis in the rodent, human, and primate genomes of fully conserved LTR7 sncRNA-encoding loci of differing lengths. Note the essentially identical profiles of genome-wide size distributions of the LTR7 sncRNA-encoding loci in the human, chimpanzee, and gibbon genomes (*C*, bottom panel; *r* = 0.998; *P* < 0.0001), with identical 24- and 18-nt sequences representing the predominantly conserved molecular entities (*A*, *D*), which are significantly overrepresented in the human genome compared with the primate and rodent genomes. Panel (*C*) (top two panels) shows evolutionary conservation in primates of chromosomal positions of the wild-type 33-nt LTR7 sncRNA-encoding sequences.

Next, a systematic survey of the genomic regulatory landscape around candidate human-specific NANOG- and CTCF-binding sites was conducted to identify regulatory elements associated with putative human-specific TF-binding events. The following genomic features of regions adjacent to human-specific TF-binding events were observed: 1) Frequent location within lamina-associated domains (LADs) (fig. 1 and supplementary fig. S2, Supplementary Material online), 2) consistent transcriptional activity (fig. 1), and 3) apparent association with domains of differential DNA methylation in hESCs (fig. 2). An example of the enrichment of TF-binding sites within LADs is illustrated in figure 1 for NANOG-binding sites embedded within full-length 6-kb LINE transposons. LADs occupy 42.9% of the human genome (Guelen et al. 2008). Therefore, based on the random distribution model, the expected number of L1-embedded NANOG-binding sites located within LADs is estimated at 72, which is significantly less than the observed number of 104 binding events located within LADs (fig. 1). A significant difference in representations between the NANOG-binding sites embedded within truncated or full-length L1 sequences and those located either within or outside the LAD boundaries was observed. Only 5% of all L1HS- and L1PA2-embedded NANOG-binding sites located within LADs are represented by truncated L1 sequences (fig. 1). In contrast, 15% of L1 retrotransposon-associated NANOG-binding events are located within truncated L1 sequences outside of the LAD boundaries (fig. 1). These data indicate that LADs represent genomic regions that favor the placement and/or retention of full-length L1 sequences harboring human-specific NANOG-binding sites. This phenomenon appears to be L1 retrotransposon-specific, because LTR7-embedded NANOG-binding events are distributed equally within and outside LADs, whereas LTR5_HS-associated NANOG-binding sites are preferentially located outside the LAD boundaries (fig. 1). Similarly, the number of human-specific RNAPII-binding sites located outside LADs is markedly enriched in both K562 and MCF cells and is depleted within LADs in both cell types (fig. 1), which is consistent with previous reports identifying LADs as chromosomal domains with a predominantly repressive chromatin environment and low transcriptional activity (Guelen et al. 2008; Peric-Hupkes et al. 2010).

Transcriptional activity is another notable feature of candidate human-specific genomic regulatory loci embedded within LINEs and LTRs. Analysis of corresponding RNA sequences in the Expression Tracks of the UCSC Genome Browser revealed that expression is not limited to established human cell lines, such as GM12878, K562, and H1-hESC. Active transcription from selected genomic loci containing human-specific TF-binding sites embedded within LINE and LTR sequences is apparent in the human brain, heart, breast, and lymph nodes. NANOG-binding sites embedded within LTR7 appear to be particularly active in H1-hESCs (fig. 1). RNA molecules were detected at nearly 40% of all LTR7-associated NANOG-binding events, and LTR7-derived transcripts account for more than 75% of all LINE- and LTR-associated transcriptional events observed in H1-hESCs (fig. 1). Consistent with these observations, Lister et al. (2009) reported that in hESCs, a subset of differentially methylated domains colocalize with dense clusters of small RNAs that map to annotated HERVs. They found that in hESCs, the HERVs were less densely methylated and were frequently associated with high downstream transcriptional activity, in contrast to the more methylated state and low proximal transcriptional activities observed in differentiated human cells (Lister et al. 2009). Alternatively, these distinct patterns of associations may be due to the differential retention of different classes of TEs in different genomic compartments. For example, the preferential retention of L1 insertion in LADs may be due to the fact that these regions consistently manifest reduced transcriptional activity that would likely reduce the potential deleterious effects caused by spurious TF binding. On the other hand, the fact that 40% of LTR7 insertions carrying NANOG-binding sites occur within transcriptionally active regions might be due to a preferential insertion of such TEs in open chromatin. Follow-up mechanistic studies will be required to distinguish between these possibilities.

### Human-Specific LADs and TF-Binding Sites Are Located within the Matrix of DNA Methylation Domains Hypermethylated in hESCs

Evidence of the frequent placement of the putative human-specific TF-binding sites within LADs suggests that at least some LADs in the human genome may constitute a component of the human-specific genomic regulatory network. To test this hypothesis, human-specific LADs were searched, which were defined as human LAD sequences which are 1) conserved in the mouse and human genomes and 2) located outside of the LAD boundaries in the mouse genome of the four distinct types of murine cells, namely mouse ESCs, neural precursor cells, astrocytes, and mouse embryonic fibroblasts (Guelen et al. 2008; Peric-Hupkes et al. 2010). Using these criteria, we identified 290 human-specific LADs representing evolutionarily conserved genomic sequences that manifest an increased propensity of association to nuclear lamina in human cells (supplementary data set S7, Supplementary Material online). Human-specific LADs constitute more than 21% of all LADs in the human genome, indicating that genome-wide interaction profiles with nuclear lamina and three-dimensional folding patterns of human chromosomes are markedly distinct. The average length of human-specific LADs is 361 kb, which is 2.7-fold shorter than the average length of all LADs in the human genome (980 kb; *P* = 5.382 E-62), and 25–40% of all LADs of nearly half of all human chromosomes are represented by the human-specific LADs (fig. 2 and supplementary fig. S3, Supplementary Material online). Unexpectedly, a significant correlation between the chromosomal distributions of 290 human-specific LADs and either 29,018 primate-specific CTCF-binding sites (*r* = 0.632; *P* = 0.0012) or 29,130 primate-specific NANOG-binding sites (*r* = 0.640; *P* = 001) was observed. In contrast, no significant correlation was found between the chromosomal distributions of 290 human-specific LADs and 14,003 primate-specific OCT4-binding sites (data not shown). These data suggest that the observed correlations cannot be explained solely by the chromosome size effects and may indicate that mechanisms of creation and retention of novel regulatory elements in the human genome are associated with common structural features. A survey of the genomic landscape in the vicinity of human-specific TF-binding sites revealed their apparent colocalization with DNA methylation sites, suggesting that genomic regions of DNA methylation may be relevant to these processes.

Next, the colocalization patterns of candidate human-specific regulatory elements with domains of differential DNA methylation in hESCs were investigated (Lister et al. 2009). Nearly 90% of human-specific LADs (257 of 290; *P* < 0.0001) were found to intersect with continuous DNA segments termed partially methylated domains (PMDs), which are hypermethylated in hESCs and hypomethylated in differentiated human IMR90 cells (Lister et al. 2009). Similarly, 90.5%, 65.1%, and 94.6% of human-specific binding events for CTCF, NANOG, and OCT4, respectively, are located within the PMDs (*P* < 0.0001 in all instances; fig. 2). This analysis demonstrates that a vast majority of the human-specific regulatory elements identified herein are located within genomic regions that are hypermethylated in hESCs and hypomethylated in differentiated human IMR90 cells. PMDs are significantly enriched for genes that are more highly expressed in hESCs compared with differentiated cells, indicating that the chromatin state within these regions in hESCs is permissive for high transcriptional activity (Lister et al. 2009). In agreement with these findings, consistent active transcription from human-specific TF-binding sites in hESCs was found, which is particularly apparent for LTR7-embedded loci (fig. 1).

A recent report demonstrates that PMDs cover 37% of the genome in the full-term human placenta (Schroeder et al. 2013), indicating that the function of these epigenetic regulatory domains is not limited to cultured cells. A total of 482 (58.4%) human-specific NANOG-binding sites were found to be located within 264 placental PMDs, documenting a statistically significant association (*P* < 0.0001) of human-specific TF-binding sites with PMDs identified in normal human tissue. Notably, among seven different tissue-specific PMDs and highly methylated domains (HMDs), only neuronal-specific domains (N-HMDs) were found to be enriched for genes located near human-specific NANOG-binding sites (2-fold enrichment; *P* = 0.0094). These data are consistent with the hypothesis that human-specific regulatory loci are preferentially placed within differentially methylated genomic regions, because N-HMD designates domains defined by sequencing of bisulfite-treated DNA (MethylC-seq) as PMDs (hypomethylated domains) in IMR90 fetal lung fibroblasts and placentas but as HMDs in SH-SY5Y neuroblastoma cells (Schroeder et al. 2013). Notably, genes located in N-HMDs were associated with synaptic transmission and neuron differentiation functions and play a role in brain and embryo development (Schroeder et al. 2013), suggesting that expression of genes of these functional categories may be affected by their close proximity to human-specific regulatory elements.

### Evolutionary Conservation in Primates of LTR7-Derived Sequences Encoding Small RNAs

Consistent with the recent reports of the increased LTR7 transcription in hESC (Kelley and Rinn 2012; Santoni et al. 2012; Xie et al. 2013; Lu et al. 2014), transcriptional activity of the LTR7-embedded human-specific NANOG-binding sites was frequently observed in hESC (fig. 1). Subsequent analytical efforts were focused on the 33- and 24-nt LTR7-derived sequences of small noncoding RNAs (LTR7 sncRNAs), which were often represented among LTR7-associated transcripts detected in hESCs (figs. 1 and 3 and supplementary fig. S4, Supplementary Material online). The initial analysis was concentrated on the genome-wide assessment of the fully conserved 33- and 24-nt LTR7 sncRNA-encoding loci, defined as the full-length sequences with 100% identity, no gaps, and no mismatches. Both sequences were classified as primate-specific, because 1) there are no full-length 33- and 24-nt LTR7 sequences in either the mouse or rat genomes, and 2) chromosomal positions for 89% of the genomic loci encoding 33-nt LTR7 sncRNAs are conserved in primate genomes, with most significant similarities noted between the human genome and those of chimpanzee and gorilla (fig. 3 and supplementary fig. S4, Supplementary Material online). Reflecting their common origin, chromosomal distributions of 33- and 24-nt sequences in the human genome manifest highly correlated patterns (*r* = 0.865; *P* < 0.0001). However, the number of loci encoding 24-nt LTR7 sncRNAs is 8.6-fold higher than that for 33-nt transcripts in the human genome (1,129 vs. 130 loci, respectively, in the hg19 human genome reference database). Additional analysis of this phenomenon is presented in the supplementary material, Supplementary Material online.

The apparent association of the LTR7 sncRNAs expressed in hESCs (fig. 1) with DNA methylation events was further explored by calculating the frequency of colocalization of sncRNA expression with methyl-cytosine in human cells (supplementary fig. S4, Supplementary Material online). Colocalization events were computed using data visualization tools in the AnnoJ browser (http://neomorph.salk.edu/human_methylome, last accessed May 20, 2015), which are designed based on a genome-wide single-base resolution human methylome map (Lister et al. 2009). I observed that 89% of loci display colocalization of DNA methylation events with sncRNA expression within the examined 200-nt-long sequences (supplementary fig. S4, Supplementary Material online). Of note, colocalization of sncRNA expression with noncanonical methylation sites at CHH or CGH (where H is any base except G) sequences appears to be significantly enriched (*P* = 0.0057; supplementary fig. S4, Supplementary Material online). Intriguingly, methylation at noncanonical methylated (m)CH sites increases most rapidly during the primary phase of synaptogenesis in the developing postnatal human brain, and a rapid mCH level increase occurs primarily in neurons (Lister et al. 2013).

### Noncoding RNA-Associated Methyl-C to T Mutation Mechanisms of Genome Editing in hESCs

It is well established that DNA methylation sites are hypermutable. The CpG sites are hypermutable because the C of CpGs is considered a preferred site of DNA methylation, and methyl-C (mC) is prone to mutate to T through spontaneous deamination (Razin and Riggs 1980; Ehrlich and Wang 1981). The net result is that CpGs are replaced over time by TpG/CpA sequences and the overall mCpG mutation rate is estimated at 10–50 times the rate of C mutation in any other context (Coulondre et al. 1978; Duncan and Miller 1980; Razin and Riggs 1980; Ehrlich and Wang 1981; Bulmer 1986; Sved and Bird 1990), or of any other base in the genome (Hwang and Green 2004). Recently, Lister et al. (2009) reported that approximately 25% of all methylation events identified in hESCs were in a non-CG context. Methylation in non-CG contexts seems specific to ESCs because non-CG methylation disappeared upon induced differentiation of the hESCs (Lister et al. 2009). These data suggest that the mutation-driving mechanism caused by the spontaneous deamination of mC to T may be relevant to the noncanonical methylation events occurring in hESCs at non-CG (e.g., CHH and CHG) sequences.

The 24-nt LTR7 sncRNA sequence contains 11 potential noncanonical methylation sites, indicating that a spontaneous mC deamination mechanism may be relevant for generation of the C to T mutations within 24-nt LTR7 sequences. This hypothesis was tested by performing a systematic search for single-site C to T mutant sequences of differing lengths in the human genome (fig. 3 and supplementary fig. S4, Supplementary Material online). Strikingly, these analyses identified 21,906 genomic loci in the human genome encoding 12- to 24-nt-long single-site C to T mutants of the 24-nt LTR7 sncRNA-encoding sequence (supplementary fig. S4, Supplementary Material online). Notably, C to T mutations at different positions within the 24-nt LTR7 sequence are generated and/or retained in the human genome with markedly different efficiency: Mutants at the C3, C4, C5, C9, and C10 sites are represented by 2,893–4,146 loci, whereas mutants at the C1, C2, C7, C8, and C11 positions are represented by 426–998 loci. The single-site C to T mutants seem to create primate-specific, predominantly highly conserved sequences because chromosomal positions of more than 90% of the examined mutant loci are conserved in the genomes of human, chimpanzee, gorilla, orangutan, and gibbon (supplementary fig. S4, Supplementary Material online). Interestingly, the generation and/or retention of mutants harboring the second C to T mutation within the same 24-nt LTR7 sequence was negligible, as relatively few loci containing double C to T mutants were found in the human genome.

Of note, multiple sequence alignment analysis of the human ESC-specific NANOG-binding sites embedded within the LTR7-derived sequences identified motif logos that appear to closely resemble previously reported consensus binding sequences of several TFs, including NANOG, POU5F1 (OCT4), GATA1, and FOXA (supplementary fig. S5, Supplementary Material online). The UA9 motif logo (supplementary fig. S5*B* and *C, *Supplementary Material online) was previously associated with TF-binding events of multiple regulatory proteins exclusively in H1-hESCs, including NANOG, BCL11A, HDAC2, ESR1, GATA2, RXRA, TCF12, and a subset of HDAC2-associated POU5F1-binding sites (Wang et al. 2012). Indeed, several nucleotide variations within the sequences resembling the diverse set of TF-binding site motif logos are consistent with the C to T and G to A patterns of mutations associated with methyl-cytosine deamination, suggesting that these mechanisms may contribute to the creation of novel TF-binding sites (supplementary fig. S5, Supplementary Material online).

Collectively, these observations suggest that mC to T mutations outside the canonical mCG sites may play an important role in continuous editing of TE-derived sequences in primate genomes. In the human genome, this mutation mechanism appears to be linked to the 25,756 DNA segments (3,850 genomic loci encoding wild-type LTR7 sncRNAs and 21,906 genomic loci encoding 12- to 24-nt-long single-site C to T mutants of the 24-nt LTR7 sncRNA) containing LTR7-derived 24-nt sequences (supplementary fig. S4, Supplementary Material online).

### Association of Candidate Human-Specific TF-Binding Sites within Brain-Specific Protein-Coding Genes with Accelerated Rates of Evolution in Primates

**Fig. 4.—:**
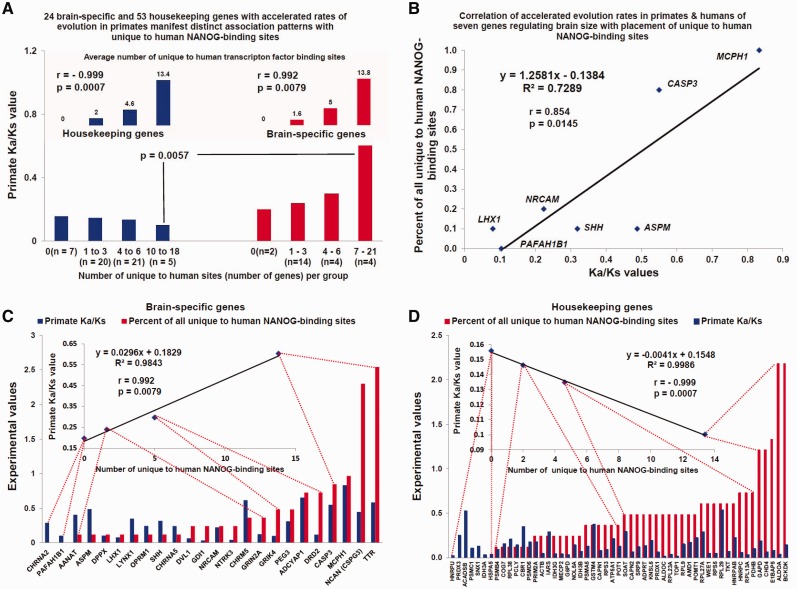
Association of human ESC-specific NANOG-binding sites with rapidly evolving protein-coding genes regulating brain size in humans. Twenty-four brain-specific and 53 housekeeping protein-coding genes that show rapid evolution in primates manifest distinct patterns of association with human-specific NANOG-binding sites (*A*, *C*, *D*). Although the placement of human-specific NANOG-binding sites is similarly enriched in close proximity to both brain-specific and housekeeping protein-coding genes (*A*, top panel; supplementary fig. S6, Supplementary Material online), the placement of human-specific NANOG-binding sites is enriched near brain-specific genes showing the highest Ka/Ks ratios (i.e., rapidly evolving genes) (*C*) and housekeeping genes (*D*) with low Ka/Ks ratios (i.e., slowly evolving genes) (*A*, bottom panel; *C*, *D*). Note the significant positive correlations between the primate Ka/Ks values and proximity placement of human-specific NANOG-binding sites for rapidly evolving protein-coding genes regulating brain size in humans (*B*).

An elite set of 24 genes regulating brain size and behavior in humans has been identified, which also shows markedly accelerated rates of protein evolution within the lineage leading from ancestral primates to humans (Dorus et al. 2004). This set of genes may play an important role in defining the phenotypic uniqueness of *Homo sapiens* by contributing to a dramatic increase in the size and complexity of the human brain during evolution (Dorus et al. 2004). Thus, it was interesting to determine how candidate human-specific TF-binding sites are positioned in the genome relative to the genomic coordinates of these 24 genes. For this analysis, the housekeeping genes were considered as the appropriate control gene set because they perform the essential basic cellular functions conserved across different species and are thus likely to evolve under evolutionary constraints without any significant impact of positive selection. Consistent with these assumptions and in contrast to the nervous system-related genes, housekeeping genes have statistically indistinguishable evolutionary rates in primates and rodents (Dorus et al. 2004). Because the set of 24 genes is characterized by a high evolutionary rate and manifests marked evolutionary rate disparities between primates and rodents (Dorus et al. 2004), the stringency of the comparison was further increased by selecting a set of 53 housekeeping genes with the highest rates of evolution in primates (fig. 4 and supplementary fig. S6, Supplementary Material online).

For each gene in both gene sets, the number of human-specific TF-binding sites with genomic coordinates showing relative proximity to protein-coding gene boundaries defined in the hg19 reference human genome database was calculated. The quantitative limits of proximity were defined based on the metrics placing human-specific TF-binding sites closer to putative target genes than experimentally defined distances to the nearest targets of 50% of the regulatory proteins analyzed in hESCs (Guttman et al. 2011). For each gene of interest, all human-specific NANOG- and CTCF-binding sites were identified with a genomic distance between TF-binding sites and a putative target gene that is smaller than the mean value of distances to the nearest target genes regulated by the protein-coding TFs in hESCs that were experimentally determined by Guttman et al. (2011).

The housekeeping genes and brain-specific genes showed similar association patterns with human-specific TF-binding sites, as measured by the number of binding events located within the boundaries of putative target gene-specific regulatory regions (table 2; fig. 4 and supplementary fig. S6, Supplementary Material online). On average, 4.00 and 3.98 human-specific TF-binding sites were located in close proximity to the rapidly evolving brain-specific and housekeeping genes, respectively. For genes in both the brain-specific and housekeeping sets, the evolutionary processes appear to favor the retention and/or creation of human-specific NANOG-binding sites over CTCF-binding sites (fig. 4 and supplementary fig. S6, Supplementary Material online). These data indicate that the overall patterns of the placement and retention of human-specific TF-binding sites are similar with respect to the genomic coordinates of either brain-specific or housekeeping genes.

**Table 2:** Association of Human ESC-Specific NANOG-Binding Sites with Brain-Specific Genes that Are Rapidly Evolving in Primates, Housekeeping Protein-Coding Genes, and Pluripotency lncRNAs

Genes	Number of Genes in the Genome	Number (%) of Genes Associated with Human-Specific NANOG-Binding Sites	Number of Associated Human-Specific TF-Binding Sites	Binding Site per Gene Ratio	Number (%) of Associated Human-Specific NANOG-Binding Sites	*P* Value^a^
Brain-specific genes	24	20 (83)	115	5.8	86 (75)	≪0.0001
Housekeeping genes	53	46 (87)	214	4.7	176 (82)	≪0.0001
Pluripotency lncRNAs	25	15 (60)	26	1.7	23 (89)	0.0059
Lineage-specification lncRNAs	34	14 (41)	27	1.9	16 (59)	0.0029^b^

^a^Hypergeometric distribution test.

^b^Less than expected by chance; <<0.0001 designates *P* = 0 at decimal place 30.

Intriguingly, this analysis revealed that brain-specific and housekeeping genes manifest remarkably different correlation profiles between the numbers of human-specific TF-binding sites and rates of protein evolution of individual genes (fig. 4 and supplementary fig. S6, Supplementary Material online). In the brain-specific gene set, a highly significant positive correlation (*r* = 0.992; *P* = 0.0079) was observed; that is, genes that are located in close proximity to more human-specific TF-binding sites manifest higher rates of protein evolution (fig. 4 and supplementary fig. S6, Supplementary Material online). In striking contrast, in the housekeeping gene set, a highly significant negative correlation (*r* =−0.999; *P* = 0.0007) was observed; that is, genes that are located in close proximity to more human-specific TF-binding sites manifest lower rates of protein evolution (fig. 4 and supplementary fig. S6, Supplementary Material online). Notably, the most highly significant correlation was observed in the brain-specific gene set between the evolutionary rates of proteins regulating brain size in humans and the numbers of human-specific NANOG-binding sites in close proximity to these genes (fig. 4 and supplementary fig. S6, Supplementary Material online). These data suggest that human ESC-specific NANOG-binding sites are located in close proximity to the rapidly evolving genes regulating the size of the human brain. In contrast, novel NANOG-binding sites appear to be preferentially located in close proximity to housekeeping genes with low rates of protein evolution. These associations are not necessarily due to the positive selective pressure. An alternative possibility is that rapidly evolving genes might be less constrained in humans and hence more tolerant to the transcriptional regulatory effect of nearby human-specific NANOG-binding sites, provided that this regulatory influence is important for neuronal gene expression. Therefore, this mechanism may be similar to the known depletion of TEs in promoters caused by the negative impact of TE insertions in genomic regulatory regions; TEs are preferentially found outside of promoter regions not because of selection for insertion in intergenic regions but rather owing to negative selection against insertion in promoters. It will be of interest to determine whether the association with rapidly evolving genes is a function of the new TF-binding sites or of TEs.

### Association of Candidate Human-specific TF-Binding Sites with Pluripotency lncRNAs and Early Developmental Enhancers

Many candidate human-specific TF-binding sites manifest hESC-specific profiles of regulatory protein binding and transcriptional activities (fig. 1). Disparities in transcriptional activities between hESCs and differentiated cells were found to be particularly evident for LTR7-derived sncRNAs (fig. 1). In contrast to protein-coding genes, TEs are embedded within 83% of human lncRNAs and comprise 42% of lncRNA sequences in the human genome (Kelley and Rinn 2012). These observations suggest that TE activity is associated with the origin, regulation, and specification of lncRNA-encoding genes (Kelley and Rinn 2012; Kapusta et al. 2013). Notably, a prominent direct correlation of the LTR7 (HERV-H) transcriptional regulatory signals with hESC-specific expression of lncRNAs has been reported (Kelley and Rinn 2012). Taken together, these observations suggest that candidate human-specific TF-binding sites, in particular, regulatory elements embedded within LTR7 sequences, may be relevant to the regulation of hESC-specific lncRNAs, namely pluripotency and lineage-specification lncRNAs (Guttman et al. 2011). Similar to the analyses performed for protein-coding genes, the number of human-specific TF-binding sites with genomic coordinates within a relative proximity to lncRNA gene boundaries defined in the hg19 reference human genome database was calculated for each lncRNA. Notably, 26 human-specific TF-binding sites were observed in close proximity to 15 of 25 pluripotency lncRNAs (supplementary fig. S6, Supplementary Material online), which is significantly higher than the number expected by chance (*P* << 0.0001). Human-specific NANOG-binding sites comprised 88% of all human-specific TF-binding events associated with pluripotency lncRNAs (table 2 and supplementary fig. S6, Supplementary Material online), whereas human-specific CTCF-binding sites were detected in only 3 of 26 instances. Conducting similar analyses for 34 lineage-specification lncRNAs (Guttman et al. 2011), we observed the association of 14 lncRNAs with 27 human-specific TF-binding sites, 16 of which are NANOG-binding sites (table 2 and supplementary fig. S6, Supplementary Material online).

The spectrum of human-specific binding events associated with lineage-specification lncRNAs appears significantly different from that of pluripotency lncRNAs (table 2 and supplementary fig. S6, Supplementary Material online). Human-specific NANOG-binding sites appear enriched near pluripotency lncRNAs (table 2): They were found in close proximity to 15 pluripotency lncRNAs, whereas CTCF-binding sites were detected in only four instances (supplementary fig. S6, Supplementary Material online). Extended analysis of these associations is presented in the supplementary material, Supplementary Material online.

Early developmental enhancers (EDEs) play important roles in human embryonic development and are distinguished in hESCs by their unique chromatin signatures and biological activities (Rada-Iglesias et al. 2011). Next, human-specific regulatory elements that are associated with the 7,596 EDEs (table 3) previously identified in hESCs (Rada-Iglesias et al. 2011) were searched. A total of 1,594 primate-specific EDEs were identified in the human genome, which is a significantly smaller number of regulatory events compared with the number of primate-specific TF-binding sites (*P* < 0.0001 in all comparisons). In contrast to TF-binding sites, only seven human-specific EDEs were found, which represents 0.09% of all EDEs in the human genome and is a marked reduction compared with the number of human-specific TF-binding events (*P* < 0.0001 in all comparisons; tables 1 and 3). Forty-six EDEs spatially associated with 50 human-specific NANOG-binding sites were identified, reflecting statistically significant enrichment of regulatory loci colocalized within 10-kb continuous genomic regions (table 3). Therefore, placement enrichment analyses revealed that human-specific NANOG-binding sites are located in close proximity to genomic coordinates of pluripotency lncRNAs and a subset of EDEs.

**Table 3:** Association of Human ESC-Specific NANOG-Binding Sites with EDEs in hESCs

EDE Classification	Number of EDEs in the Genome^a^	Number (%) of Primate-Specific EDEs	Number of Human-Specific EDEs	Number^b^ of EDEs Close to Human-Specific NANOG-Binding Sites	P Value^c^	Number of EDE-Associated Human-Specific NANOG-Binding Sites	*P* Value^c^
Class I	5,116	1,157 (22.6)	6	31	1.89 E-06	36	9.21 E-09
Class II	2,285	421 (18.4)	1	14	0.000873	13	0.002296
Class II–I	195	16 (8.2)	0	1	0.291112	1	0.291112
Total	7,596	1,594 (21)	7	46	8.15 E-09	50	1.29 E-10

^a^Genomic coordinates and classification of the EDEs were reported in Rada-Iglesias et al. (2011).

^b^Number of enhancers and binding sites with overlapping genomic coordinates within continuous 10-kb regions.

^c^Hypergeometric distribution test.

### Association of Human ESC-Specific NANOG-Binding Sites with Functional Distal hESC Enhancers and 5-Hydromethylcytosine

The results of the present analysis suggested that one of the potential mechanisms of the bioactivity of human-specific NANOG-binding sites may be associated with the functions of promoter-distal regulatory elements. To test this hypothesis, the colocalization pattern of human-specific NANOG-binding sites and distal regulatory elements was assessed utilizing a comprehensive database of hESC enhancers (Xie et al. 2013). A total of 431 colocalization events were observed between 264 human-specific NANOG-binding sites and 331 hESC enhancers within 10-kb continuous DNA segments in the hESC genome. The placement enrichment metrics near hESC-restricted enhancers were significantly higher compared with those of all hESC enhancers; the enrichment values were increased by 2.28-fold for human-specific NANOG-binding sites, 1.69-fold for hESC enhancers, and 1.71-fold for colocalization events. Furthermore, we found that 39 human-specific NANOG-binding sites are co-localized with hESC enhancers that are implicated with 122 high-confidence enhancer/protein-coding gene regulatory pairs.

**Fig. 5.—:**
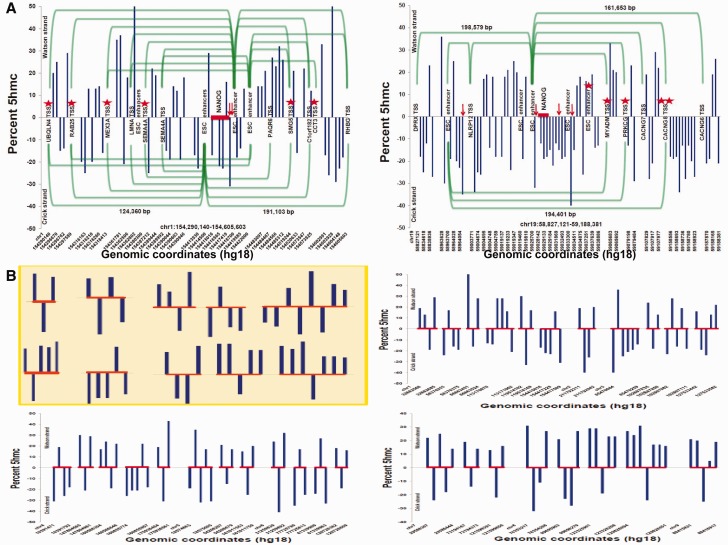
hESC genomic maps of NANOG-binding sites, adjacent 5hmC, and functional hESC enhancer/gene pairs involved in enhancer-coding gene interactions. Snapshots of base-resolution 5hmC maps in the H1-hESC genome in close proximity to the human-specific NANOG-binding sites located near functional hESC enhancers and involved in high-confidence enhancer-target gene interactions, as reported by Xie et al. (2013). Only predicted enhancer-gene pairs with a *P* value ≤ 0.0001 were considered in the analysis. Also shown are genomic positions of the human-specific NANOG-binding sites (horizontal red bars), hESC enhancers, transcription start sites (TSS) of enhancer-targeted protein-coding genes, and maps of hESC enhancer-gene interaction pairs in H1-hESC (semitransparent green arcs). Positive blue bar values indicate the 5hmC content on the (+) Watson strand of DNA, whereas negative values indicate the 5hmC content on the (−) Crick strand. For 5hmC values, the vertical axis limits are − 50% to + 50%. Note that human-specific NANOG-binding sites (red horizontal bars marked by the NANOG signs) are placed in the heart of the large-scale hESC regulatory domains near functional distal enhancers within high-complexity multidimensional regulatory networks of interacting enhancer-gene pairs (*A*) as well as within bidirectional (supplementary fig. S7, Supplementary Material online) distal regulatory domains. Examples of 5hmC patterns identified near human-specific NANOG-binding sites on all human chromosomes are shown in (*B*) (see also supplementary fig. S7, Supplementary Material online). The exact genomic positions of 5hmC are indicated by blue vertical bars, NANOG-binding sites coordinates are depicted by red horizontal bars (unique to human loci), red arrows (primate-specific sequences), and red stars (sites common with rodents) based on the hg18 release of the human genome reference database. Note that human- and primate-specific NANOG-binding sequences are located near hESC enhancers, whereas NANOG-binding sites common with rodents are placed next to the TSS of protein-coding genes. Examples of common 5hmC three- to eight-letter symbols utilized to create a large variety of 5hmC patterns near human-specific NANOG-binding sites are shown in the yellow box (*B*, top left panel).

Functional distal regulatory elements in hESCs are markedly enriched for 5-hydroxymethylcytosine (5hmC), which is most enriched in regions immediately adjacent to sequence motifs of TF-binding sites (Yu et al. 2012). Using a base-resolution map of 5hmC in the hESC genome, I determined that 5hmC is located near 46% and 21% of human-specific NANOG-binding sites within 1-kb and 100-bp windows near human-specific NANOG-binding sites, respectively (fig. 5 and supplementary fig. S7, Supplementary Material online). In contrast, no enrichment of 5hmC nucleotides near human-specific CTCF-binding sites within 1-kb windows was observed. Only 15% of CTCF-binding sites were located near 5hmC, which is significantly lower than the number of human-specific NANOG-binding sites (*P* < 0.0001). Similarly, no significant enrichment of 5hmC nucleotides was observed near human-specific POU5F1-binding sites. Only 14.9% of POU5F1-binding sites were located within 1 kb of 5hmC, which is significantly lower than the number of colocalization events documented for human-specific NANOG-binding sites (*P* < 0.0001). These data indicate that a large number of human-specific NANOG-binding sites harbor 5hmC within the canonical CpG context immediately adjacent to TF-binding sequence motifs, because in hESCs nearly all (99.89%) 5hmCs exist in the canonical CpG context (Yu et al. 2012). Additional details of this analysis are reported in the supplementary material, Supplementary Material online.

Collectively, these observations suggest that candidate human-specific TF-binding sites are most likely to play a functional role in hESCs at promoter-distal regulatory elements. This hypothesis was confirmed by building regulatory maps of selected genomic regions depicting all NANOG-binding sites, hESC enhancer/coding gene regulatory pairs, and 5hmC distributions near regulatory loci (fig. 5 and supplementary fig. S7, Supplementary Material online). Interestingly, human- and primate-specific NANOG-binding sequences were found to be located near hESC enhancers, whereas NANOG-binding sites common with rodents were located next to the transcription start site of protein-coding genes (fig. 5*A* and supplementary fig. S7, Supplementary Material online).

Analysis of 5hmC sequences located near human-specific NANOG-binding sites revealed common patterns created by the frequent usage of particular sequences, examples of which are depicted in figure 5*B* and supplementary figures S7 and S8, Supplementary Material online. In particular, a three-letter 5hmC symbol with one window in the center of a sequence due to asymmetrical placement of the middle 5hmC on the opposite DNA strand has been detected particularly often. Indeed, 57% of sequences containing at least three 5hmC nucleotides and harboring at least one 5hmC adjacent to human-specific NANOG-binding sites within 100-bp windows contain this particular symbol (fig. 5*B* and supplementary figs. S7 and S8, Supplementary Material online). In total, 81 instances of colocalization of at least one copy of this three-letter 5hmC symbol in sequences containing multiple 5hmC nucleotides within 1-kb windows centered at 826 human-specific NANOG-binding sites were observed (supplementary figs. S7 and S8, Supplementary Material online). This number is likely an underestimate because only high-abundance 5hmC sequences are detectable at the reported sequencing depth and significantly greater sequencing is required to obtain high-confidence resolution of low-abundance 5hmC sequences at the single-base level (Yu et al. 2012). In contrast to human-specific NANOG-binding sequences, only eight 5hmC sequences within 1-kb windows near 591 human-specific CTCF-binding sites contain this three-letter 5hmC symbol, and the NANOG protein was bound to four of these sites (*P* < 0.0001). Similarly to CTCF-binding sites and in sharp contrast to human-specific NANOG-binding sequences, only 20 events displaying this three-letter symbol of 5hmC sequences were observed within 1-kb windows near 2,386 human-specific POU5F1-binding sites, and NANOG protein was bound to 13 of these sites (*P* < 0.0001).

Taken together, these data reveal association of human ESC-specific NANOG-binding sites with functional hESC enhancers and suggest that the common patterns of 5hmC sequences identified here may contain a code to signal recruitment of NANOG protein to specific genomic loci.

### Human ESC-Specific NANOG-Binding Sites Are Located in Close Proximity to Developmentally- and Pathophysiologically Associated Coding Genes

To test whether coding genes located in close proximity to human ESC-specific NANOG-binding sites have pathophysiological associations or important developmental roles, the Ingenuity Pathway Analysis software was utilized (http://www.ingenuity.com/, last accessed May 20, 2015). The Ingenuity Pathway Analysis of coding genes that have human-specific NANOG-binding sites within gene bodies or near gene boundaries identified a core set of 135 genes that appear to be interconnected in multiple networks associated with nervous system development and functions, embryonic development, behavior, and cardiovascular system development and functions (supplementary data set S8 and video, Supplementary Material online). One of the notable features of this set of genes is their apparent association with development of a broad spectrum of common human disorders, including cancer, cardiovascular diseases, reproductive system diseases, metabolic diseases, multiple neurological and psychological disorders, hereditary and developmental diseases, and many other disease states (supplementary data set S8 and video, Supplementary Material online). Taken together, these data argue that human ESC-specific NANOG-binding sites may contribute to regulation of coding genes with important physiological roles in development of nervous and cardiovascular systems during embryogenesis.

### Evolutionary Aspects of TE Contribution to the Emergence of Novel Regulatory Elements

One of the limitations of the analyses conducted so far is that they were performed using the reference genome databases and not the individual genomes. To address this limitation, the assessment of conservation of 826 human ESC-specific NANOG-binding sites in individual genomes of three Neanderthals and five Modern Humans was carried-out by direct comparisons of corresponding sequences retrieved from individual genomes and the human genome reference database (http://genome.ucsc.edu/Neandertal/, last accessed May 20, 2015).

**Fig. 6.—:**
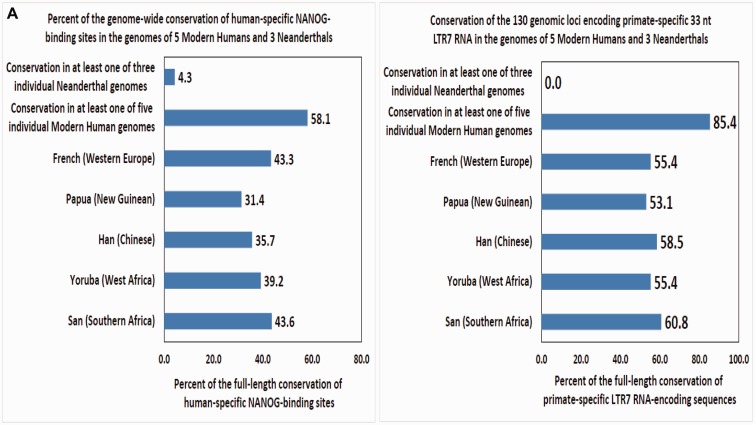

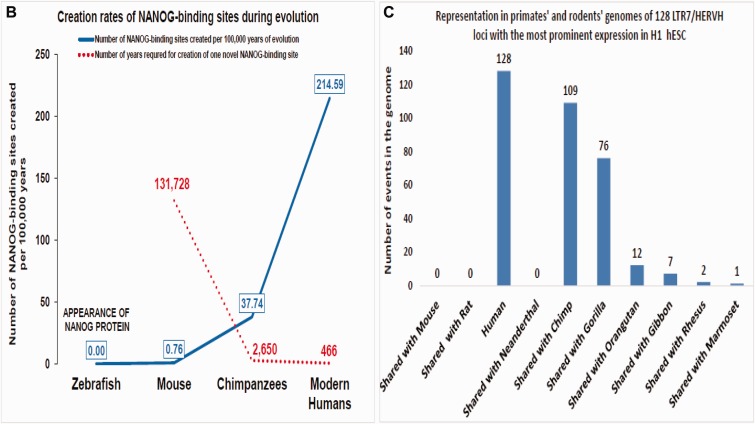

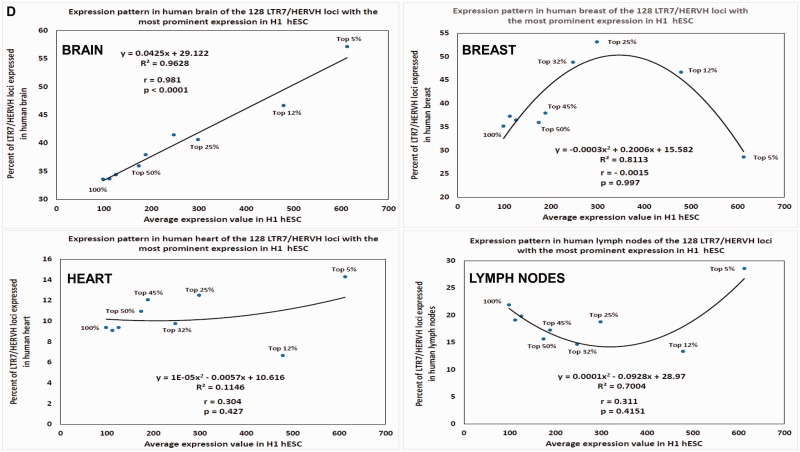
Patterns of conservation (*A*, *C*), expression (*D*), and creation during evolution (*B*) of human ESC-specific NANOG-binding sites (*A*, *B*) and LTR7/HERVH sequences (*A*, *C*, *D*) in individual genomes of five Modern Humans and three Neanderthals. (*A*) Patterns of the full-length sequence conservation of 826 human ESC-specific NANOG-binding sites (left panel) and 130 genomic loci encoding 33 nt LTR7 RNA (right panel) in genomes of five Modern Humans and three Neanderthals. (*B*) Estimates of creation rates of novel NANOG-binding sites during evolution. See table 4 and text for further details. (*C*) Representation in primates’ and rodents’ genomes of 128 LTR7/HERVH genomic loci with the most prominent expression in the H1 hESC cells. (*D*) Expression patterns in adult human tissues of 128 LTR7/HERVH genomic loci with the most prominent expression in the H1 hESC cells.

This analysis reveals an interindividual variability in degree of conservation of human-specific NANOG-binding sites ranging from 31.4% to 43.6% in individual genomes of Modern Humans. Conservation of full-length human-specific NANOG-binding sequences was 31.4% in Papua (New Guinean), 35.7% in Han (Chinese), 39.2% in Yoruba (West Africa), 43.3% in French (Western Europe), and 43.6% in San (Southern Africa). The sequence homology analysis identifies 152 sequences (18.4%) that are conserved in all five individual human genomes, whereas 58.1% of human-specific NANOG-binding sites are conserved in at least one individual genome of Modern Humans (fig. 6). In striking contrast, only 32 sequences (4.3%) of human ESC-specific NANOG-binding sites are conserved in at least one Neanderthals genome, suggesting that a majority of human ESC-specific NANOG-binding sites (95.7%; 794 sequences) emerged in Modern Humans after the Modern Humans/Neanderthals split approximately 370,000 years ago (Noonan et al. 2006). Emergence in genomes of Modern Humans of 794 regulatory sequences during 370,000 years implies that 2.15 novel NANOG-binding sites evolved in hESC genome every 1,000 years or one novel NANOG-binding site per 466 years of evolution (table 4; fig. 6).

**Table 4:** Estimates of Creation Rates of Novel TF-Binding Sites during Evolution

Species	TFs	Number of Novel TF-Binding Sites	Number of New TF-Binding Sites per 100,000 Years	Number of Years Required to Create One New Binding Site	Chimp/Mouse and Human/Chimp Ratios of Creation Rates
Mouse^a^	NANOG	2,657	0.76	131,718	
Chimpanzee^b^	NANOG	28,304	37.74	2,650	49.7
Modern Humans^c^	NANOG	794	214.59	466	5.7
Chimpanzee	CTCF	28,427	37.90	2,638	
Modern Humans	CTCF	591	159.73	626	4.2
Chimpanzee	POU5F1	11,617	15.49	6,456	
Modern Humans	POU5F1	2,386	644.86	155	41.6
Chimpanzee	RNAPII	11,693	15.59	6,414	
Modern Humans	RNAPII	319	86.22	1,160	5.5

^a^Calculated based on estimates of approximately 350-Myr evolutionary timeline between Zebrafish and Mouse.

^b^Calculated based on estimates of Humans and Chimpanzees split 13 Ma and 88 Myr from Euarchonta and Glires (Gliriformes) split resulting in the estimated evolutionary timeline of approximately 75 Myr.

^c^Calculated based on estimates of Modern Humans and Neanderthals split 370,000 years ago; estimates for CTCF, POU5F1, and RNAPII were calculated based on the assumption that all human-specific TF-binding sites emerged after Modern Humans and Neanderthals split 370,000 years ago; estimates of creation rates of primate-specific TF-binding sites were based on the assumption that all primate-specific TF-binding sites emerged before Modern Humans/Chimpanzees split 13 Ma.

The reported numbers of NANOG-binding sites in zebrafish, mouse, and human genomes are 14,010, 16,667, and 88,351, respectively (Chen et al. 2008; Kunarso et al. 2010; Xu et al. 2012), suggesting that creation of NANOG-binding sites was markedly accelerated during primate’s evolution. The estimates of creation rates of TF-binding sites in the rodent’s genomes indicate that 131,728 years were required for creation of one novel NANOG-binding site during approximately 350 Myr of evolution from Zebrafish to Mouse (table 4 and fig. 6). Analysis of the creation rates of primate-specific NANOG-binding sites reveals that 2,650 years were required for creation of one novel NANOG-binding site (table 4 and fig. 6) during approximately 75 Myr of evolution from Euarchonta and Glires split approximately 88 Ma to Chimpanzees/Modern Humans split approximately 13 Ma (Venn et al. 2014). Taken together, this analysis suggests that there was 49.7-fold acceleration of creation rates of NANOG-binding sites in genomes of Chimpanzees compared with the mouse genomes and further 5.7-fold acceleration in genomes of Modern Humans compared with the Chimpanzees genomes (table 4 and fig. 6).

Similar results were obtained during the analysis of human-specific and primate-specific CTCF-binding sites indicating that creation rates of novel CTCF-binding sites were 4.2-fold higher in humans compared with Chimpanzees (table 4). Assuming that all human-specific TF-binding sites emerged after Modern Humans/Neanderthals split, rates of creation of novel RNAPII-binding sites appear 5.5-fold higher in Modern Humans compared with Chimpanzees, requiring for emergence of one new RNAPII-binding sites 1,160 and 6,414 years of evolution in humans and great apes, respectively (table 4). There was a 41.6-fold acceleration of the emergence of novel POU5F1-binding sites in Modern Humans compared with Chimpanzees (table 4). The creation of one novel POU5F1-binding sites during evolution of Modern Humans and Chimpanzees required 155 and 6,456 years, respectively (table 4). Validation of these observations by direct sequencing and follow-up functional analyses of candidate primate-specific and human-specific regulatory sequences will be required.

Analysis of rates of creation of novel primate-specific and human-specific TF-binding sites indicates that there was a marked acceleration of novel TF-binding site creation in genomes of Chimpanzee and Modern Humans compared with rodents, which may have been associated with the increased activity of primate-specific retrotransposons during evolution. Consistent with this hypothesis, analysis of sequence conservation of 130 genomic loci encoding LTR7 RNAs in individual genomes of three Neanderthals and five modern humans reveals that 1) no LTR7 RNA-encoding sequences are present in Neanderthals’ genomes and 2) overall evolutionary conservation patterns of 130 LTR7 RNA-encoding loci and 826 human ESC-specific NANOG-binding sites are strikingly similar (fig. 6). In contrast to Neanderthals genomes, there are 34 full-length LTR7 RNA-encoding loci in the 41,000-year-old Denisovan genome (Reich et al. 2010; Meyer et al. 2012).

Several additional lines of experimental evidence suggest that likely candidate retrotransposons with markedly accelerated activity may be found among HERVs, in particular, LTR7/HERVH family. The LTR7 subfamily is rapidly demethylated and upregulated in the blastocyst of human embryos and remains highly expressed in human ES cells (Smith et al. 2014). In human ESC and iPSC, LTR7 sequences harboring the promoter for the downstream full-length HERVH-int element, as well as LTR7B and LTR7Y sequences, were expressed at the highest levels and were the most statistically significantly upregulated retrotransposons (Fort et al. 2014). LTRs of HERV-H, in particular, LTR7, function in hESC as enhancers and HERVH sequences encode nuclear noncoding RNAs, which are required for maintenance of pluripotency and identity of hESC (Lu et al. 2014). Transient hyperactivation of HERVH is required for reprogramming of human cells toward iPSC, maintenance of pluripotency, and reestablishment of differentiation potential (Ohnuki et al. 2014). Failure to control the LTR7/HERVH activity leads to the differentiation-defective phenotype in neural lineage (Koyanagi-Aoi et al. 2013; Ohnuki et al. 2014). Kunarso et al. (2010) identified LTR7/HERVH as one of the most overrepresented TEs seeding NANOG- and POU5F1-binding sites throughout the human genome. Expression of HERVH appears regulated by the pluripotency regulatory circuitry as 80% of LTRs of the 50 most highly expressed HERVH are occupied by pluripotency core TFs, including NANOG and POU5F1 (Santoni et al. 2012). The continuing activity of L1 retrotransposons may also contribute to the accelerated rate of creation of primate-specific TF-binding sites during evolution because significant activities of both L1 transcription and transposition were recently reported in humans and other great apes (Marchetto et al. 2013).

In human ESC, 128 LTR7/HERVH loci with markedly increased transcription were identified (Lu et al. 2014). These genomic loci represent the most likely functional candidates from the LTR7/HERVH family playing critical regulatory roles in maintenance of pluripotency and transition to differentiation phenotypes in humans. Conservation analysis of the 128 LTR7/HERVH loci with the most prominent expression in hESC demonstrates that none of them is present in Neanderthals genome, whereas 109 loci (85%) are shared with Chimpanzee (fig. 6). Considering that Neanderthals’ genomes are approximately 40,000 years old and Chimpanzee’s genome is contemporary, these results are in agreement with the hypothesis that LTR7/HERVH viruses were introduced in primates’ population approximately 40,000 years ago. Distinct patterns of expression of different subsets of transcripts selected from 128 LTR7/HERVH loci hyperactive in hESC are readily detectable in adult human tissues, including various regions of human brain (fig. 6). These observations support the idea that sustained LTR7/HERVH activity may be relevant to physiological functions of human embryos and adult human organs, specifically human brain.

Analysis of estimated creation rates of novel TF-binding sites during evolution indicates that there was a marked acceleration of TF-binding site creation in primates’ genomes compared with rodent. This preliminary analysis reveals that estimated creation rates of novel NANOG-binding sites appear increased approximately 283-fold in the ESC genomes of Modern Humans compared with the Mouse ESC genome. During the most recent evolutionary period this phenomenon seems linked to the increased activity of HERVs, in particular, LTR7/HERVH family.

### A Proximity Placemen Enrichment Model of the Open Chromatin State Maintenance

At the nanoscale level, genomic DNA molecules are highly dynamic. DNA double helix continually acquires a transitory protein-less state due to highly dynamic nature of interactions with nucleosome core and linker histones. Methylation-associated DNA editing (MADE) alters the conventional double helix by replacing Watson–Crick base pairs with less energetically efficient noncanonical base pairing, creating nucleotide mismatches and nucleotide evictions at selected sites (Lee J, Hwang M, Landon PB, Lal R, Glinsky GV, in preparation). Recent experiments have shown that structural changes of DNA sequences resembling MADE markedly enhance the responsiveness of DNA to invading DNA and noncoding RNA molecules (Lee J, Hwang M, Landon PB, Lal R, Glinsky GV, in preparation) and these properties of “edited” DNA double helix can be exploited to engineer next-generation nanosensors, DNAzymes, and DNA motors (Glinsky 2013; Landon et al. 2014; Mo et al. 2014). Invading RNAs induce a thermodynamically stable partial strand displacement state (PSDS), which may persist indefinitely as triple-stranded complexes (TSC) comprising RNA/DNA hybrids and single-stranded DNA chains. In contrast, conventional DNA double helix did not respond to invading RNA, failed transition to PSDS, and did not acquired the TSC conformation.

Energetic biases introduced by MADE change kinetics of strand displacement reactions to favor creation of a thermodynamically-stable PSDS with single-stranded DNA exposed for interactions with regulatory proteins for extended time periods. Importantly, more than 90% of TF-binding sites, including binding sites for NANOG, POU5F1, and CTCF, are represented by the single-stranded DNA sequences (Wang et al. 2012). Therefore, non-coding RNA-induced transition of DNA double helix to a triple-stranded conformation with exposed single-stranded DNA sequences may represent a default regulatory state of protein-less DNA interacting with invading RNA molecules within the open chromatin architecture (Lee J, Hwang M, Landon PB, Lal R, Glinsky GV, in preparation). Interactions with TFs would stabilize a partial strand displacement conformation, thus impeding the nucleosome formations, resisting the compaction of nucleosome arrays and interfering with the packaging of DNA chains into condensed, repressed chromatin. Conversely, DNA chains within repressed chromatin architecture would manifest decreased responsiveness to invading RNA molecules, slow kinetics of DNA strand displacement, and decreased likelihood of acquisition of a PSDS of DNA double helix (Lee J, Hwang M, Landon PB, Lal R, Glinsky GV, in preparation).

According to this model, sequence-specific interactions of LTR7 non-coding RNAs with DNA would induce thermodynamically-stable PSDSs and transition to triple-stranded DNA/RNA hybrid complexes with exposed single-stranded DNA chains. Binding of single-strand-specific TFs, such as NANOG, POU5F1, and CTCF, to the exposed single-stranded DNA sequences would lead to stabilization of a PSDS and maintenance of the open chromatin architecture in the vicinity of selected genomic loci. TEs are primary targets of MADE, because DNA methylation plays crucial roles in the inactivation of transposons to protect mammalian genomes from their harmful mutational activity (Yoder et al. 1997). According to the Repeatmasker algorithm, there are 3,523 LTR7 elements in the human genome (Koyanagi-Aoi et al. 2013). Therefore, biological roles of transcriptionally active LTR7/HERVH sequences may be associated with distal regulatory effects of noncoding LTR7 RNA molecules contributing to the maintenance of open chromatin architecture at thousands genomic loci throughout human genome.

The proximity placement enrichment model does not require that all primate- and human-specific TF-binding sites function in a manner similar to the classic promoter, enhancer, repressor, or insulator elements. This indicates that 33–47% of the excess NANOG and/or POU5F1 proteins (table 1) that are immobilized on a DNA scaffold may affect the chromatin state and transcriptional regulatory landscape in selected genomic regions. For example, the large excess of TF-binding sites at selected genomic locations in primate genomes is likely to dramatically influence the three-dimensional chromatin conformation and affect transitions between distinct regulatory states, as postulated in the “strings and binders switch” model of chromatin dynamics (Barbieri et al. 2012). Placement enrichment of primate- and human-specific TF-binding sites at selected genomic regions may facilitate chromatin remodeling and transition to the permissive chromatin states (Ernst and Kellis 2013), enabling binding of multiple regulatory proteins and cobinding of many TFs at specific loci (supplementary figs. S9–S11, Supplementary Material online). Consistent with this model, statistically significant placement enrichment of human-specific NANOG-binding sites in close proximity to the genomic coordinates of protein-coding genes regulating brain size that are rapidly evolving in primates, pluripotency lncRNAs, functional hESC enhancers, and EDEs was observed.

## Discussion

TEs contributed to the creation of thousands new species-specific TF-binding sites in mammals (Wang et al. 2007; Bourque et al. 2008; Kunarso et al. 2010). Computational and bioinformatics analyses of DNase I hypersensitivity sites in more than 40 distinct human cell types demonstrated that nearly two-thirds of primate-specific open chromatin regions were in TEs (Jacques et al. 2013), indicating that a majority of novel regulatory elements in the primate lineage evolved within TEs. Consistently, current analysis identifies in human genome 84,163 primate-specific TF-binding sites for NANOG, CTCF, POU5F1, and RNAPII proteins (table 1), only a small fraction of which represents human-specific regulatory elements. Notably, 64% of candidate human-specific NANOG-binding sites are located within LTR/LINE-derived sequences (table 1), in contrast to 17.6%, 4.9%, and 1.6% of human-specific TF-binding sites for CTCF, POU5F1, and RNAPII (table 1). These data indicate that creation of candidate human-specific regulatory loci with putative TF-binding sites for different regulatory proteins is associated with distinct families of TEs and may be driven by mechanistically distinct evolutionary processes. LTR/LINE-associated mechanisms are of particular interest due to the recent evidence of both transcriptional and transposition activities of these TEs in hESC (Garcia-Perez et al. 2007; Macia et al. 2011; Kelley and Rinn 2012; Santoni et al. 2012; Wissing et al. 2012; Xie et al. 2013), human embryos (Smith et al. 2014), iPSC of humans and great apes (Marchetto et al. 2013), and brain tissues from individual humans (Baillie et al. 2011).

Phylogenetic conservation can be considered as strong evidence for an important functional role of genetic regulatory elements. However, as species-specific regulatory events are by definition not conserved, it is difficult to differentiate the biological significance of such events from mere background noise without conducting detailed functional experiments. However, a total of 84,163 identified herein primate-specific sequences harboring putative TF-binding sites are present in the reference genome databases of humans and chimpanzees, suggesting that they were created before the Modern Humans/Chimpanzees split 13 Ma and conserved during divergent evolution of Modern Humans and Chimpanzees. Genome-wide proximity placement analyses of 826 human ESC-specific NANOG-binding sites revealed apparently nonrandom placement and significant enrichment near distinct genomic elements, including PMDs, N-HMDs, functional hESC enhancers, enhancer/coding gene regulatory pairs, asymmetrical 5hmC sites, pluripotency lncRNAs, EDEs, and protein-coding genes regulating brain size in humans that show rapid evolution among primates. Multiple sequence alignment analysis identified motif logos of the TE-embedded NANOG-binding sites, which closely resemble previously reported consensus binding sequences of the POU5F1 (OCT4) and NANOG TFs (supplementary fig. S5, Supplementary Material online). These data provide a relatively short list of defined genetic loci, including candidate human-specific regulatory elements and their putative genomic targets, which may be functionally linked and should be useful for design of follow-up mechanistic experiments.

Identification of thousands of primate- and human-specific sequences that appear to function as protein-specific binding elements in ESCs raises the question regarding the potential functional impact of this large-scale chromatin rewiring in the human genome. One intriguing possibility is that this phenomenon occurs owing to the extended time of transcriptional control of the pluripotency factors NANOG and POU5F1 at selected genomic loci during early developmental stages. Dynamic local concentrations of the NANOG and POU5F1 proteins near chromatin regions enriched in TF-binding sites would be maintained at the biologically effective threshold levels for longer time periods during the gradual decline of NANOG and POU5F1 expression in embryos. Notably, many TF-binding-enriched loci contain protein-coding genes that were implicated in regulation of critical elements of nervous system development such as the quantity, density, and functionality of neurons, axons, synapses, and neurotransmitter receptors.

The results of the present analysis indicate that candidate human-specific NANOG-binding sites are enriched within PMDs that are highly methylated in hESCs and N-HMDs highly methylated in neuronal cells, suggesting that neuronal-specific HMDs harboring synaptic transmission and neuron differentiation genes are established during the early embryonic stages of brain development. In agreement with this hypothesis, human ESC-specific NANOG-binding sites were found to be enriched near 5hmC sequences within the canonical CpG context immediately adjacent to TF-binding sequence motifs. Several lines of experimental evidence support this model. In the human frontal cortex, 5hmC appears to be more abundant in neurons than glial cells (Lister et al. 2013). Frontal cortex development is accompanied by increased enrichment of 5hmC at intragenic regions that are already hyperhydroxymethylated at the fetal stage, demonstrating that adult patterns of genic 5hmC in the frontal cortex are already evident in the immature fetal brain (Lister et al. 2013). In the human brain, overall transcriptional activity is associated with intragenic 5hmC enrichment, with in utero establishment of adult 5hmC patterns for cell type-specific genes, and loss of 5hmC enrichment is associated with developmentally coupled transcriptional downregulation (Lister et al. 2013).

According to previous reports, TF-binding sites associated with repetitive elements constitute only a relatively small fraction of all binding events. For example, repeat-associated binding sites accounted for 11.1% and 28.3% of all CTCF-binding events in genomes of human and mouse ESCs, respectively (Kunarso et al. 2010). Similarly, only 15% of 88,351 NANOG-binding sites and 22% of 29,740 OCT4-binding events in hESCs are associated with repeats (table 1). In this study, a remarkably high association was observed between human-specific TF-binding sites and repetitive elements; essentially all (99.8%; 3,797 of 3,803) candidate human-specific regulatory sequences are embedded within repeats. Therefore, this study provides further support for the idea that TEs represent a major evolutionary force for creation of new genomic loci with regulatory functions (Wang et al. 2007; Bourque et al. 2008; Kunarso et al. 2010) and highlights the potential mechanistic links between DNA methylation-associated genome editing and emergence of candidate human-specific TF-binding sites.

The present analysis supports the hypothesis that genome-editing mechanisms associated with active (autonomous) and passive retrotransposition, DNA methylation of TE-derived sequences, and mC deamination and hydroxylation may facilitate the emergence of human-specific regulatory networks. The placement and/or retention of candidate human-specific NANOG-binding sites within regulatory networks seems to favor close proximity to the genomic coordinates of protein-coding genes regulating brain size that are rapidly evolving in primates, pluripotency lncRNAs, functional hESC enhancers, and a subset of EDEs (tables 2 and 3; figs. 4 and 5 and supplementary fig. S6, Supplementary Material online). These results suggest that genomic regions that are hypermethylated in hESCs (PMD domains) may provide a permissive chromatin environment for emergence of human-specific regulatory sites. PMD domains are significantly enriched in genes that are overexpressed in hESCs compared with differentiated cells, indicating that the chromatin state within these regions is permissive for high transcriptional activity in hESCs (Lister et al. 2009). Present analysis suggests that the permissive chromatin state within these regions may be associated with the increased activity of LTR7/HERVH elements and maintained by the LTR7 RNAs. Collectively, these data support the hypothesis that human-specific TF-binding sites function at distal regulatory elements within networks regulating nervous system development during embryogenesis. Targeted gain- and loss-of-function follow-up experiments are required to determine whether these human-specific genomic regulatory networks actually exist and operate. In this regard, experimental analyses of potential functional connectivity between candidate human-specific TF-binding sites and brain-specific protein-coding genes with markedly accelerated evolution in the primate ancestor to human lineage are of particular interest.

This study provides possible answers to important questions as to why and how TE-derived sequences become functional DNA in the human genome. Several distinct mechanisms of mutations within repeat-derived sequences may facilitate the LINE and LTR transposon-driven creation of the NANOG-, OCT4-, and CTCF-binding sites at new genomic locations. The intrinsic property of reverse transcriptase to generate a high error rate when transcribing RNA into DNA likely represents one of the major sources of mutations as, unlike any other DNA polymerases, reverse transcriptase has no proofreading ability. The intrinsically high error rate of reverse transcription associated with active and passive retrotransposition coupled with DNA methylation and mC deamination and hydroxylation would facilitate accumulation of mutations at an accelerated rate, thus providing a molecular basis for the emergence and disappearance of novel TF-binding sites. It is tempting to speculate that continuing cycles of LTR7 activity may operate within this mechanistic context as exquisite, primate-specific designers and erasers of regulatory elements at new genomic locations.

CpG methylation and deamination play crucial roles in the inactivation of transposons to protect mammalian genomes from their harmful mutational activity (Yoder et al. 1997). Methylation and deamination of CpGs embedded within Alu transposons in the human genome resulted in the generation of thousands of p53-binding sites with the preferred core motif composed of CpA and TpG dinucleotides (Zemojtel et al. 2009). Indeed, CpG deamination events may create TF-binding sites with much higher efficiency than other single nucleotide mutational events (Zemojtel et al. 2009). Evolutionary analysis of TF-binding sites in ESCs is consistent with the idea that CpG deamination is a major contributor to the creation of novel binding sites for NANOG, OCT4, and CTCF (Kunarso et al. 2010; Zemojtel et al. 2011). These naturally occurring genome-editing mechanisms may play an important biological role in human evolution by markedly increasing the combinatorial regulatory complexity of individual genomes and enhancing the phenotypic diversity of individual cells within populations. Transient activation of these mechanisms in embryogenesis may contribute to the development of individually unique profiles of performance, fitness, longevity, and adaptation potential of multicellular organisms during ontogeny.

## Conclusions

Identification and initial characterization of candidate human-specific regulatory elements revealed that TEs and cytosine MADE contribute to creation of thousands novel TF-binding sites in human genomes. Analysis of individual genomes of Neanderthals and Modern Humans indicates that a majority of human ESC-specific NANOG-binding sites emerged after the Modern Humans/Neanderthals split. Creation rates of novel TF-binding sequences appear markedly accelerated during the evolution of Modern Humans and Chimpanzees compared with rodents, which may have been associated with activation of primate-specific retrotransposons. Consistent with their functional role in ESC, a total of 84,163 primate-specific TF-binding sequences for NANOG, POU5F1. CTCF, and RNAPII proteins were continuously created and retained during the evolution after the Euarchonta and Glires split approximately 88 Ma. Coding genes located in close proximity to human-specific TF-binding sites are associated with physiological development and functions of nervous and cardiovascular systems, embryonic development, behavior, as well as development of a broad spectrum of common human diseases, including multiple neurological and psychological disorders. Location of human ESC-specific NANOG-binding sites appears significantly enriched near distinct genomic elements, including PMDs, N-HMDs, functional hESC enhancers, enhancer/coding gene regulatory pairs, asymmetrical 5hmC sites, pluripotency lncRNAs, EDEs, and protein-coding genes regulating brain size in humans that show rapid evolution among primates. Possible regulatory mechanisms affecting the chromatin state and transcriptional landscape in selected genomic regions of hESC involve activation of LTR7 transcription and genome-wide distal regulatory effects of LTR7 RNAs. Availability of iPSC from humans and other great apes (Marchetto et al. 2013) makes it feasible to undertake in the near future direct experimental analyses of the functional role of identified herein candidate human-specific regulatory elements.

## Supplementary Material

Supplementary text, data sets S1–S8, figures S1–S11, table S1, and video are available at *Genome Biology and Evolution* online (http://www.gbe.oxfordjournals.org/).
